# State-level household gun ownership proxy dataset, 1949–2020

**DOI:** 10.1016/j.dib.2023.109548

**Published:** 2023-09-07

**Authors:** Megan Kang, Elizabeth Rasich

**Affiliations:** a Department of Sociology, Princeton University, 107 Wallace Hall, Princeton, NJ 08544, United States; b Crime Lab, The University of Chicago, 33 N. La Salle, Ste. 1600, Chicago, IL 60602, United States

**Keywords:** Firearm proxy, Household gun ownership rates, Firearm suicide, Firearm homicide

## Abstract

This dataset expands an existing proxy for U.S. household gun ownership rates, known as the rate of firearm suicides divided by all suicides (FSS), covering 1949–2020, with newly added data for the 1949–1972 period. For each state and year, the dataset provides the count and population-adjusted rate of suicides, firearm suicides, homicides, and firearm homicides, among other figures. The first 30 years of firearm suicide/homicide counts were transcribed from scanned National Center for Health Statistics reports; later figures come from the CDC's WONDER and WISQARS systems. Unlike other measures of gun prevalence that focus on national or regional variation, this proxy captures trends in household gun ownership at the state level. Moreover, it does not rely on self-reported data, which are susceptible to social desirability bias. To the best of our knowledge, this extended proxy dataset represents the longest-ranging collection of state-level gun ownership rates available to date. By utilizing the FSS proxy, researchers can examine long-term patterns and changes in household gun ownership rates. This dataset also opens up opportunities to explore the effects of gun ownership on public health, social dynamics, policy, and other relevant areas of research.

Specifications Table

**Table:** 

Subject	Public health and health policy; Criminology
Specific subject area	State-level household gun ownership proxy (1949–2020) State-level gun homicide and suicide data (1949–2020)
Type of data	Table
How the data were acquired	Firearm suicide, homicide and firearm homicide counts for 1949–1978 were transcribed by hand from National Center for Health Statistics scans of Vital Statistics reports. They were merged with firearm and suicide counts from the CDC's Web-based Injury Statistics Query Reporting System (WISQARS) from 1981 to 2020, and from the CDC's (Wide-ranging Online Data for Epidemiologic Research) WONDER for 1979 and 1980.
Data format	The data are in raw format and have been analyzed. An Excel file with data has been uploaded.
Description of data collection	The U.S. government began collecting state-level firearm suicide data in 1949, and encoded the data by gender, race, and state. We transcribed firearm suicide counts and suicide counts and then calculated the rate of suicides committed with a firearm to create the FSS proxy. To construct firearm homicide and firearm suicide rates per 100,000 residents, we drew population data from linearly interpolated decennial Census population counts from 1948 to 2005 and 2020, and from the American Community Survey from 2006 to 2019.
Data source location	Primary source data were gathered from CDC WISQARS and WONDER, Center for Disease Control and Prevention's National Center for Health Statistics, Atlanta, GA. The data were analyzed at the Department of Sociology, Princeton University, Princeton, NJ.
Data accessibility	Harvard Dataverse: Firearm Suicide Proxy for Household Gun Ownership, 1949–2020, https://doi.org/10.7910/DVN/QVYDUD Direct URL to data: https://dataverse.harvard.edu/dataset.xhtml?persistentId=doi:10.7910/DVN/QVYDUD
Related research article	M.S. Kang, E.A. Rasich, Extending the Firearm Suicide Proxy for Household Gun Ownership. Am. J. Prev. Med. (2023) doi:https://doi.org/10.1016/j.amepre.2023.05.003

## 1 Value of the Data

• This dataset [1] provides the most extensive proxy for state-level household gun ownership rates, spanning from 1949 to 2020, with new coverage for the period between 1949 and 1972.
• Researchers in various fields, including medical sciences, public health, social sciences, and public policy, can benefit from these data to gain insights into the factors influencing gun ownership and the impact of gun prevalence on public health over time.
• The state-level variation captured by this gun prevalence proxy allows for finer-grain detail and inferences. The lack of data from sub-regional units is a limitation of many gun survey measures, inhibiting research that examines how state-level changes affect gun ownership, such as changes in state policies and demographic trends.
• The proxy captures racial variation, enabling researchers to study how social changes, such as the Great Migration or civil rights movements in the 1960s, have affected gun ownership for specific racial groups.
• The proxy suggests that the significant and prolonged increase in household gun ownership may have begun earlier than previously believed. Further investigation is needed to explore these trends, but it is plausible that the rise in gun ownership is an unexpected outcome of the economic prosperity following World War II [2].

## 2 Objective

The U.S. has more guns than people and more than double the rate of guns per capita than the next highest country, Yemen [3]. The vast majority of homicides in the U.S. are also committed with a gun. As we researched how and why the U.S. came to have so many guns, we realized there were no reliable historical sources of data on gun prevalence. Data for what is considered the best available household gun ownership proxy—firearm suicides as a percentage of total suicides (FS/S or FSS)—had only been validated and made public from 1973 onward. That meant availability of the best proxy for gun ownership did not start until well after the mid-20th century spike in homicides began in the 1960s. This, combined with evidence that relatively fewer homicides prior to the 1960s spike were committed with guns and polling suggesting Americans’ reasons for owning a gun had shifted over time, prompted us to fill in this substantial gap in order to better understand what happened in American gun culture and usage between the mid-20th century and today.

## 3 Data description

There are two files in the repository. The first is the “Firearm_suicide_homicide_codebook.csv”, which describes the variables and sources of the data. The variables included in this dataset are also outlined in Table 1 below.

**Table 1:** Variables description.

Variable name	Variable description	Source
State	State name (includes Washington, D.C.)	
Year	Year	
Division	Census division	
Total_population	Linearly interpolated decennial Census population counts	from 1949 to 2005 and 2020 and from the American Community Survey from 2006 to 2019.
fss	Firearm suicide divided by total suicide (FSS)	1949–1978 National Center for Health Statistics scans of Vital Statistics reports; 1981–2020 from CDC WISQARS; 1979 and 1980 from CDC WONDER
Homicide_rate	Homicide rate per 100,000 residents	1949–1978 National Center for Health Statistics scans of Vital Statistics reports; 1981–2020 from CDC WISQARS; 1979 and 1980 from CDC WONDER
Firearm_homicide_rate	Firearm homicide rate per 100,000 residents	1949–1978 National Center for Health Statistics scans of Vital Statistics reports; 1981–2020 from CDC WISQARS; 1979 and 1980 from CDC WONDER
Nonfirearm_homicide_rate	Firearm homicide rate per 100,000 residents	1949–1978 National Center for Health Statistics scans of Vital Statistics reports; 1981–2020 from CDC WISQARS; 1979 and 1980 from CDC WONDER
Firearm_suicides	Count of firearm suicides	1949–1978 National Center for Health Statistics scans of Vital Statistics reports; 1981–2020 from CDC WISQARS; 1979 and 1980 from CDC WONDER
Total_suicides	Count of all suicides	1949–1978 National Center for Health Statistics scans of Vital Statistics reports; 1981–2020 from CDC WISQARS; 1979 and 1980 from CDC WONDER
Firearm_homicides	Count of firearm homicides	1949–1978 National Center for Health Statistics scans of Vital Statistics reports; 1981–2020 from CDC WISQARS; 1979 and 1980 from CDC WONDER
Nonfirearm_homicides	Count of all nonfirearm homicides	1949–1978 National Center for Health Statistics scans of Vital Statistics reports; 1981–2020 from CDC WISQARS; 1979 and 1980 from CDC WONDER
Total_homicides	Count of total homicides	1949–1978 National Center for Health Statistics scans of Vital Statistics reports; 1981–2020 from CDC WISQARS; 1979 and 1980 from CDC WONDER
White_fss	FSS for white population	1949–1978 National Center for Health Statistics scans of Vital Statistics reports; 1981–2020 from CDC WISQARS; 1979 and 1980 from CDC WONDER
Nonwhite_fss	FSS for nonwhite population	1949–1978 National Center for Health Statistics scans of Vital Statistics reports; 1981–2020 from CDC WISQARS; 1979 and 1980 from CDC WONDER
Nextyearfss	Next year's FSS	1949–1978 National Center for Health Statistics scans of Vital Statistics reports; 1981–2020 from CDC WISQARS; 1979 and 1980 from CDC WONDER
Nextyearnonwhitefss	Next year's nonwhite FSS	1949–1978 National Center for Health Statistics scans of Vital Statistics reports; 1981–2020 from CDC WISQARS; 1979 and 1980 from CDC WONDER
Nextyearwhitefss	Next year's white FSS	1949–1978 National Center for Health Statistics scans of Vital Statistics reports; 1981–2020 from CDC WISQARS; 1979 and 1980 from CDC WONDER

The second file in the repository is the “Firearm_suicide_homicide_dataset.tab”, which provides a state-level measure of household gun prevalence known as FSS (firearm suicides / total suicides) from 1949 to 2020, with new coverage for the 1949–1972 period. It also includes the count and population-adjusted rate of suicides, firearm suicides, homicides, and firearm homicides, among other figures, for the sample period.

WISQARS suppresses suicide counts under 10 for 1999–2019 so there are fewer observations available for that time. Suppressed data were filled in through 1) requesting causes of death for other reasons, 2) requesting causes of death for the same reasons but excluding gun deaths, and then 3) doing basic algebra to get the suppressed counts. For example: Causes of death (include firearms and motor vehicle) - Cause of death (motor vehicle) = firearm deaths.

Summary statistics are presented in Table 2 below. Note that in multiple instances the total number of recorded suicides by a particular racial group was zero, mechanically leading to a case where FSS could not be observed. (This was particularly acute in low-population states.) Note also that Alaska became a U.S. state in 1959, but we draw population data from interpolated decennial Censuses; because Alaska was not a state in 1950, we only have population data for the state starting in 1960. This also means we are unable to calculate a homicide rate, firearm homicide rate, or non-firearm homicide rate for Alaska in 1959.

**Table 2:** Summary statistics.

## 4 Experimental design, materials and methods

To overcome the limitations of administrative and self-reported data on gun prevalence, researchers have historically used indirect measures to estimate rates of gun ownership. One widely used indirect measure involves calculating the ratio of suicides committed with a firearm (FS) to total suicides (S), denoted as FSS. This proxy has been regarded as a reliable indicator of U.S. households that possess at least one firearm [4], [5], [6]. However, prior to our study, the validity of FSS as a proxy for the proportion of American households with firearms had only been established starting from 1973.

In our companion paper, Kang and Rasich (2023), we confront the methodological challenge of validating a proxy for gun ownership during a timeframe in which reliable data on firearm prevalence is lacking [2]. Our aim was to evaluate whether FSS accurately represents household gun ownership rates at the state level spanning the years 1949–1972. Our validation strategy involved collating historical firearm-related data and undertaking a sequence of statistical tests with FSS during this earlier period. While no individual assessment offered conclusive proof independently, the collective results of these evaluations provided compelling evidence of the dependability of FSS as a proxy for household firearm ownership rates from 1949 to 1972. In this section, we provide an overview of the research methods we employed to validate this extended proxy for household gun ownership rates.

### 4.1 Homicide data

Due to the unavailability of state-level gun ownership surveys or indicators for the period spanning 1949–1972, we adopted an alternative approach by examining homicide and firearm homicide rates to validate the FSS proxy. Prior studies have demonstrated a strong correlation between gun ownership and homicide, particularly firearm homicide rates, at the state level [7], [8], [9]. In our validation, we digitized homicide and firearm homicide counts per state from 1949 to 1968, as these data were not previously accessible through CDC WONDER or WISQARS online. Population estimates from the Census were utilized to compute state-level homicide and firearm homicide rates, reaching back to 1949.

To gauge the consistency of this relationship across time, we ran panel regressions within 15-year intervals, where firearm homicide and homicide rates were regressed against FSS. In line with past panel studies that examine shifts in state-level gun ownership over time within the United States, our analysis included state and year fixed effects into the analysis to determine the relationship between homicides and FSS. The outcomes of these regression analyses, presented in Appendix Tables 1 and 2 within Kang and Rasich (2023), consistently revealed positive and statistically significant coefficients across time periods. This persistent pattern indicates a strong and reliable relationship between FSS and gun ownership during preceding decades, thereby aligning with our expectations if FSS serves as a dependable substitute for actual rates of household gun ownership.

### 4.2 Firearm shipments

We also examined the relationship between FSS and firearm shipments spanning the years 1949–1998. The Bureau of Alcohol, Tobacco, and Firearms (ATF) provide annual records of gun shipments, starting from 1946. These records represent the annual flow of new firearms into the domestic market, categorized by firearm type. While the gun shipment data doesn't extend to the state level, it stands as one of the few consistent indicators of firearms that stretches back to 1949. Consequently, it offers a valuable means for confirming the validity of FSS across an extensive timeframe.

Firearm shipment data are indirectly related to the underlying construct of household gun ownership rates, for which FSS serves as a proxy. An increase in gun shipments may or may not directly result in an increase in household gun prevalence, as it depends on who is purchasing the guns. For instance, if the majority of new guns are purchased by households that already own guns (referred to as the ‟intensive margin”), then gun shipments would increase while FSS would remain constant. On the other hand, if the new guns are primarily bought by households that do not currently own guns (referred to as the ‟extensive margin”), both gun shipments and FSS would increase. If FSS is a reliable proxy for household gun ownership rates and the increase in guns between 1949 and 1998 is predominantly driven by growth at the extensive margin, we would expect to observe a relationship between domestic gun shipments and FSS during these years.

In Figure 2 of Kang and Rasich (2023), we presented the relationship between national gun shipments and FSS. Since FSS serves as a proxy for household gun stock while the ATF data captures total gun flows, we compared the two measures in terms of year-over-year changes. This analysis enabled us to identify similar trends in the year-over-year changes of FSS and handgun shipments between 1949 and 1998. This finding further bolstered our confidence in the reliability of FSS in earlier decades. Additionally, we standardized the trend lines using z-scores and observed a highly consistent growth rate between ATF handgun shipments and FSS rates over time (for further details, see Appendix Figure 1 in Kang and Rasich (2023)).

### 4.3 Survey measures

We also investigated the correlation between FSS and national survey data on household gun ownership rates. While surveys have their limitations, such as historical gaps in coverage, biases related to self-reporting, and limited accuracy at the state level, they are one of the few available sources of information during the period we are validating the FSS proxy. As a result, they provide invaluable insights for evaluating FSS.

To create a time series of survey measures, we collected data from the General Social Survey, which is considered a gold standard national survey on gun ownership rates and is available from 1973 onwards. We also gathered all other national surveys that asked about household gun ownership prior to 1973. These early surveys were obtained from Cornell University's Roper Center for Public Opinion Research. The earliest survey in our sample dates back to 1959. In total, we identified only nine surveys that met our criteria, some of which were conducted in the same year. We then calculated the average gun ownership rate for each year, resulting in a single indicator based on the early survey measures.

Within Figure 3 of Kang and Rasich (2023), we presented the trend line of FSS alongside the composite survey indicator and RAND's measurement (HFR) for the years that were available. This comparison revealed a close correspondence between the composite survey indicator and FSS from 1959 to 1972, with noticeable divergence starting in 1973. If FSS accurately reflects gun ownership during this earlier period, we would expect to observe a positive association between FSS and survey-based measures of household gun ownership. The close alignment between the composite survey measure and FSS during the examined years further supports the reliability of FSS as a proxy for actual household gun ownership rates.

### 4.4 Stability over time

Lastly, we examined the cross-sectional relationship between FSS and other gold-standard measures of gun prevalence over time available beginning 1973. If we observed a consistent and stable relationship between FSS and these alternative indicators, it would provide greater confidence in extrapolating this stability to the period where we lack direct validation measures.

To test this, we computed the bivariate correlation coefficients between FSS with two supplementary measures: the General Social Survey (GSS) evaluations of household gun ownership and a new measure known as Household Firearm Ownership Rates (HFR), developed by Schell et al. (2020) at the RAND Corporation [10]. The HFR measure offers a state-level estimate of household gun ownership, available from 1980 to 2016. It combines various survey measures and proxies of gun prevalence to gauge the proportion of households with a gun for each state between 1980 and 2016.

In Figure 4 of Kang and Rasich (2023), we exhibited the bivariate correlation coefficients across different time points between FSS and both the GSS gun prevalence measures and HFR. The results showed consistently robust and steady correlations between FSS and the GSS measure, as well as FSS and HFR, spanning the years 1973–2020. Based on these findings, we extrapolated the stability in the relationship between FSS and actual gun ownership rates from 1949 to 1972. The persistent and strong cross-sectional correlations between FSS and the other reliable indicators provide further support for the reliability of FSS as a proxy for household gun ownership rates during this earlier period.

## Ethics statement

This work does not involve human subjects, animal experiments, or data collected from social media platforms. We did not need permission for using the primary data.

## CRediT authorship contribution statement

**Megan Kang:** Conceptualization, Methodology, Validation, Investigation, Data curation, Writing – original draft, Writing – review & editing, Visualization, Supervision. **Elizabeth Rasich:** Conceptualization, Investigation, Data curation, Writing – original draft, Project administration.

## Data Availability

Firearm Suicide Proxy for Household Gun Ownership, 1949-2020 (Original data) (Dataverse).
